# Differential aging‐related changes in neurophysiology and gene expression in IB4‐positive and IB4‐negative nociceptive neurons

**DOI:** 10.1111/acel.12795

**Published:** 2018-06-25

**Authors:** Malgorzata A. Mis, Mark F. Rogers, Aaron R. Jeffries, Anna L. Wilbrey, Lubin Chen, Yang Yang, Sulayman Dib‐Hajj, Stephen G. Waxman, Edward B. Stevens, Andrew D. Randall

**Affiliations:** ^1^ School of Physiology, Pharmacology, and Neuroscience University of Bristol Bristol UK; ^2^ Intelligent Systems Laboratory University of Bristol Bristol UK; ^3^ University of Exeter Medical School University of Exeter Exeter UK; ^4^ Pfizer Neuroscience and Pain Research Unit Cambridge UK; ^5^ Department of Neurology and Center for Neuroscience and Regeneration Research Yale University School of Medicine New Haven Connecticut USA; ^6^ Rehabilitation Research Center Veterans Administration Connecticut Healthcare System West Haven Connecticut USA; ^7^ Department of Medicinal Chemistry and Molecular Pharmacology Purdue University College of Pharmacy and Purdue Institute for Integrative Neuroscience West Lafayette Indiana USA; ^8^ Institute of Biomedical and Clinical Sciences University of Exeter Medical School Hatherly Laboratories University of Exeter Exeter UK

## Abstract

Despite pain prevalence altering with age, the effects of aging on the properties of nociceptors are not well understood. Nociceptors, whose somas are located in dorsal root ganglia, are frequently divided into two groups based on their ability to bind isolectin B4 (IB4). Here, using cultured neurons from 1‐, 3‐, 5‐, 8‐, 12‐, and 18‐month‐old mice, we investigate age‐dependent changes in IB4‐positive and IB4‐negative neurons. Current‐clamp experiments at physiological temperature revealed nonlinear changes in firing frequency of IB4‐positive, but not IB4‐negative neurons, with a peak at 8 months. This was likely due to the presence of proexcitatory conductances activated at depolarized membrane potentials and significantly higher input resistances found in IB4‐positive neurons from 8‐month‐old mice. Repetitive firing in nociceptors is driven primarily by the TTX‐resistant sodium current, and indeed, IB4‐positive neurons from 8‐month‐old mice were found to receive larger contributions from the TTX‐resistant window current around the resting membrane potential. To further address the mechanisms behind these differences, we performed RNA‐seq experiments on IB4‐positive and IB4‐negative neurons from 1‐, 8‐, and 18‐month‐old mice. We found a larger number of genes significantly affected by age within the IB4‐positive than IB4‐negative neurons from 8‐month‐old mice, including known determinants of nociceptor excitability. The above pronounced age‐dependent changes at the cellular and molecular levels in IB4‐positive neurons point to potential mechanisms behind the reported increase in pain sensitivity in middle‐aged rodents and humans, and highlight the possibility of targeting a particular group of neurons in the development of age‐tailored pain treatments.

## 1 INTRODUCTION

According to the United Nations Population Division, the number of people aged 60 or over is expected to increase by 56% by 2030 (UN, 2015). Middle‐aged and older people are more likely to both report painful conditions and receive analgesic treatments, with the prevalence of certain types of pain peaking at midlife (Buskila, Abramov, Biton, & Neumann, 2000; Rustoen et al., 2005; Schopflocher, Taenzer, & Jovey, 2011; Yeo & Tay, 2009). While this is partly due to the higher prevalence within these groups of age‐related diseases, such as diabetes, cancer, arthritis, and stroke (Ayis, Gooberman‐Hill, & Ebrahim, 2003; Denton & Spencer, 2010), healthy aging affects pain mechanisms at both peripheral and central levels (Farrell, 2012; Yezierski, 2012) and is likely to contribute to the reported statistics on age dependence of pain prevalence.

Indeed, animal models show that aged mice are at a higher risk of developing neuropathic pain following a spared nerve injury (Bishay et al., 2013), and aged rats suffer significantly longer from injury‐induced thermal hyperalgesia (Lovell, Novak, Stuesse, Cruce, & Crisp, 2000) and tactile allodynia (Crisp, Giles, Cruce, McBurney, & Stuesse, 2003). Interestingly, some rodent pain models also point to increased pain sensitivity in midlife. For example, 18‐month‐old rats exhibited a higher sensitivity to pain induced with formalin injection than 3‐ and 24‐month‐old rats (Gagliese & Melzack, 1999), and a longitudinal study on mice showed that middle‐aged animals are most sensitive to pain‐inducing electrical stimuli (Finkel et al., 2006). At the primary sensory neuron level, studies showed a decreased central projection of myelinated neurons (Bergman & Ulfhake, 2002), a decrease in substance P and calcitonin gene‐related peptide expression, and an increase in neuropeptide Y expression in dorsal root ganglia (DRG) neurons from aged rats (~30 months; Bergman, Johnson, Zhang, Hokfelt, & Ulfhake, 1996), suggesting that the differences in pain behaviors are likely contributed to by aging‐related changes in the properties of nociceptors (i.e., sensory neurons activated by painful stimuli).

Nociceptors can be classified into two broad neurobiochemical groups based on their ability to bind isolectin B4: IB4‐positive (IB4+) and IB4‐negative (IB4−) neurons (Silverman & Kruger, 1990; Stucky & Lewin, 1999). IB4+ and IB4− neurons have been shown to be different subsets at the transcriptome level (Chiu et al., 2014), follow different peripheral (Zylka, Rice, & Anderson, 2005) and central (Braz, Nassar, Wood, & Basbaum, 2005) pathways, and have been suggested to be involved in the development and maintenance of different types of pain, with IB4+ neurons implicated in mechanical and chronic pain, and IB4− neurons in inflammatory and acute pain (Dirajlal, Pauers, & Stucky, 2003; Malmberg, Chen, Tonegawa, & Basbaum, 1997; Mantyh et al., 1997).

Here, we combine whole‐cell patch‐clamp and RNA‐seq techniques to assess a range of age‐dependent alterations in neurophysiology and transcriptome of mouse IB4+ and IB4− DRG neurons and show that the two groups of neurons “age” in distinct ways. While the excitability of IB4− neurons was found to be relatively age‐independent, the excitability of IB4+ neurons followed an inverted “U” pattern with a peak in firing rate at 8 months, suggesting that IB4+ neurons may contribute to higher susceptibility to pain around middle age, and pointing to the possibility of targeting this subset of nociceptors in treatments of age‐dependent chronic and neuropathic pain.

## 2 RESULTS

### 2.1 Excitability of IB4+ but not IB4− neurons displays pronounced age‐dependent changes

**Figure 1:**
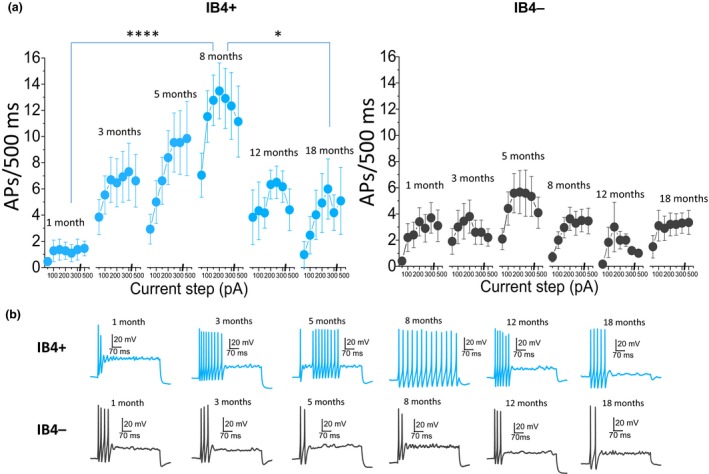
Age dependence of firing properties of IB4+ and IB4− dorsal root ganglia (DRG) neurons. (a) Average number of action potentials (APs) fired in response to incremental 500 ms current steps. Left panel: Data for IB4+ neurons. The effect of age is statistically significant (*F* = 12, *p* < 0.001, one‐way repeated‐measures ANOVA;* n*: 1 m = 11, 3 m = 13, 5 m = 11, 8 m = 22, 12 m = 6, 18 m = 13) with neurons from 8‐month‐old mice firing more APs than neurons from the youngest (*p* = 0.0001) and oldest (*p* = 0.02) mice (Bonferroni post hoc test). Right panel: Data for IB4− neurons. The effect of age is not statistically significant (*F* = 0.9, *p* = 0.5, one‐way repeated‐measures ANOVA;* n*: 1 m = 10, 3 m = 10, 5 m = 11, 8 m = 24, 12 m = 6, 18 m = 20). (b) Example traces show voltage responses to 500 ms 300 pA current injections for IB4+ and IB4− DRG neurons from mice of different ages

Firing frequency of DRG neurons has been shown to be strongly linked to pain mechanisms, with an increase in firing rate indicative of an increase in pain intensity (Devor, 2006; Zhang et al., 2013). We studied age dependence of the firing rate of IB4+ and IB4− DRG neurons, by inducing action potentials (APs) with 500 ms depolarizing current steps at 35°C. Unexpectedly, an inverted “U” pattern emerged within the IB4+ group of neurons, with the cells from 8‐month‐old mice firing the largest number of spikes, as can be seen in Figure 1 (*F* = 12, *p* < 0.001, one‐way repeated‐measures ANOVA; 1–8 m *p* = 0.0001; 8–18 m *p* = 0.02; Bonferroni post hoc test). In contrast, no changes were observed in the IB4− group of cells (*F* = 0.9, *p* = 0.5, one‐way repeated‐measures ANOVA), pointing to pronounced differences in age dependence of excitability between IB4+ and IB4− neurons. To decipher the mechanisms behind these differences in firing rate, we studied the effect of aging on two major determinants of neuronal excitability: resting membrane potential (RMP) and input resistance (Ri). High Ri increases the electrogenic sensitivity of neurons, with stimulations of set intensities resulting in larger voltage change. Together with depolarized RMP, which brings neurons closer to AP threshold, it would be expected to increase neuronal excitability. For clarity, we focused on IB4+ neurons from the age groups where significant differences in the firing rate were found (1, 8, and 18 months), as well as their IB4− counterparts.

**Figure 2:**
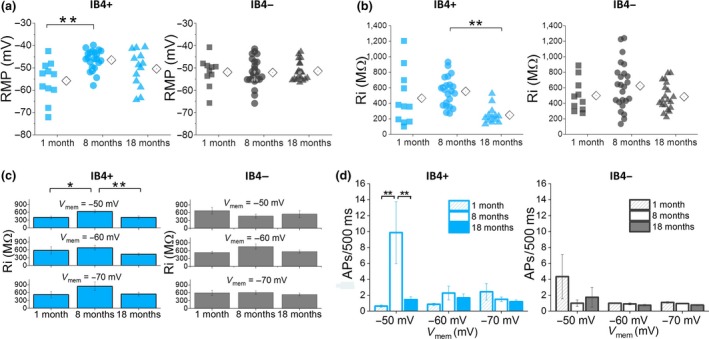
Age dependence of resting membrane potential (RMP) and input resistance (Ri) of IB4+ and IB4− dorsal root ganglia neurons. (a) RMP data for IB4+ and IB4− neurons. Each symbol represents a single cell; to the right of each scatter is the mean (diamond symbol). The effect of age is significant for IB4+ (*F* = 7, *p* = 0.003, one‐way ANOVA;* p* = 0.002, Bonferroni post hoc test), but not IB4− neurons (*F* = 0.1, *p* = 0.9, one‐way ANOVA). (b) Ri values for IB4+ and IB4− neurons. The effect of age was significant within the IB4+ (*F* = 8, *p* = 0.001, one‐way ANOVA;* p* = 0.001; Bonferroni post hoc test), but not the IB4− group (*F* = 2, *p* = 0.1, one‐way ANOVA). (c) Left panel: Data for IB4+ neurons showing the average Ri at different set prestimulus potentials (*F* = 6, *p* = 0.003, two‐way ANOVA, 1–8 m *p* = 0.04, 8–18 m *p* = 0.001; Bonferroni post hoc test). Right panel: Data for IB4− neurons (*F* = 0.4, *p* = 0.7, two‐way ANOVA). (d) Average number of action potentials (APs) in response to a 500 ms 500 pA current step. Left panel: Data for IB4+ neurons. Cells from 8‐month‐old animals fired significantly more APs at −50 mV (*F* = 3.3, *p* = 0.04, two‐way repeated‐measures ANOVA; 1–8 m *p* = 0.003, 8–18 m *p* = 0.008, Bonferroni post hoc test; *n*: 8–22). Right panel: Data for IB4− cells. No significance was found here (*F* = 0.3, *p* = 0.8, two‐way repeated‐measures ANOVA;* n*: 12–28)

Resting membrane potential values for individual IB4+ and IB4− neurons are shown in Figure 2a. The effect of age was significant only within the IB4+ group (1 m = −56 ± 2 mV, 8 m = −47 ± 1 mV, 18 m = −51 ± 2 mV; *F* = 7, *p* = 0.003, one‐way ANOVA), with the average RMP of neurons from 8‐month‐old mice significantly more depolarized than the average RMP of neurons from 1‐month‐old mice (*p* = 0.002, Bonferroni post hoc test). Mean RMP values for the IB4− neurons did not differ between the three ages (1 m = −52 ± 2 mV, 8 m = −53 ± 1 mV, 18 m = −52 ± 1 mV; *F* = 0.1, *p* = 0.9, one‐way ANOVA). Also, age was found to have a significant impact on Ri in IB4+, but not IB4− neurons, as shown in Figure 2b (IB4+: 1 m = 463 ± 92 MΩ, 8 m = 525 ± 39 MΩ, 18 m = 245 ± 31 MΩ; *F* = 8, *p* = 0.001, one‐way ANOVA; IB4−: 1 m = 506 ± 57 MΩ, 8 m = 618 ± 68 MΩ, 18 m = 452 ± 34 MΩ; *F* = 2, *p* = 0.1, one‐way ANOVA). Post hoc tests revealed significant differences between IB4+ cells from 8‐ and 18‐month‐old mice (*p* = 0.001; Bonferroni post hoc test), again pointing to more pronounced age‐dependent changes in IB4+ than IB4− DRG neurons. As Ri is dependent on membrane potential, it was also studied at set prestimulus potentials of −50, −60, and −70 mV. Columns in Figure 2c present the average Ri values for IB4+ (left panel) and IB4− (right panel) neurons at the three potentials. There was a significant age effect within the IB4+ group (*F* = 6, *p* = 0.003, two‐way ANOVA), with cells from 8‐month‐old mice displaying the highest average Ri at all membrane potentials (*p* < 0.05, Bonferroni post hoc test). In contrast, no significant interaction was observed between age and Ri within the IB4− group (*F* = 0.4, *p* = 0.7, two‐way ANOVA).

Firing frequency of both groups of neurons was also evaluated at set membrane potentials using the same series of incremental 500 ms current steps employed in Figure 1. For clarity, only the average number of APs fired in response to 500 pA current steps is presented in Figure 2d. Unexpectedly, while the effect of age was significant again within the IB4+ group (*F* = 3.3, *p* = 0.04; two‐way repeated‐measures ANOVA), the inverted “U” pattern found at RMP (Figure 1) was only observed at prestimulus membrane potential of −50 mV. On the other hand, IB4− neurons displayed a similar pattern of firing at all three potentials (Figure 2d right panel; *F* = 0.3, *p* = 0.8; two‐way repeated‐measures ANOVA). This finding likely points to the presence of proexcitatory conductances activated at depolarized membrane potentials that, together with a significantly higher Ri, lead to an increase in excitability of IB4+ neurons from 8‐month‐old mice. These differences in the impact of age on excitability of IB4+ and IB4− DRG neurons point to differences in age‐dependent gene expression between the two groups of cells.

### 2.2 RNA‐seq data point to varied age‐dependent transcriptome changes

**Figure 3:**
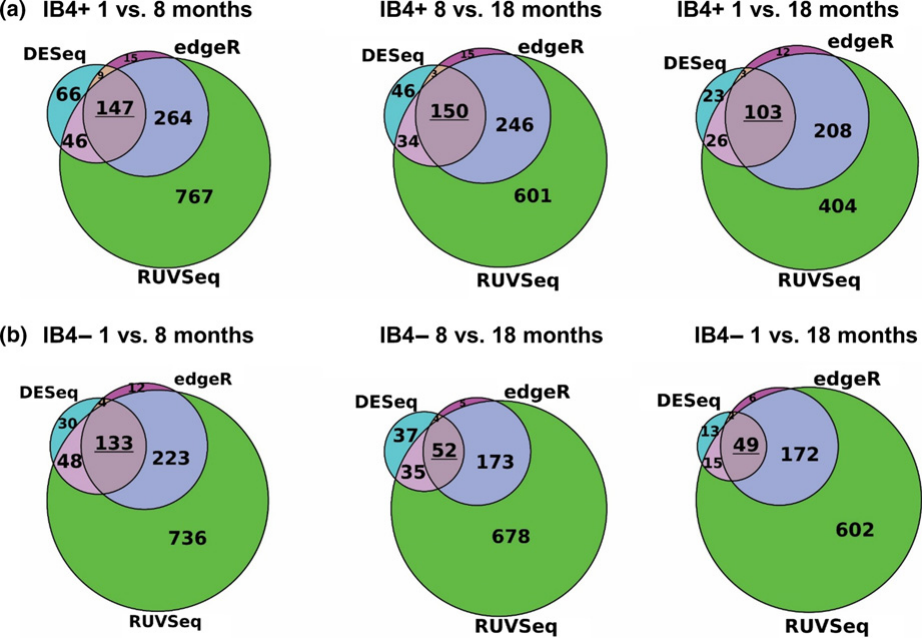
Differentially expressed (DE) analysis from DESeq, edgeR, and RUVSeq reveals genes that may be differentially regulated over time in IB4+ and IB4− neurons. Selected were genes that were significant (*p* ≤ 0.01) for all three DE prediction methods from 1 to 8 months and from 8 to 18 months and again from 1 to 18 months. This yielded 147, 150, and 103 genes for IB4+ (a) and 133, 52, and 49 genes for IB4− neurons (b)

To determine the effect of aging at the molecular level, we conducted RNA‐seq experiments on IB4+ and IB4− DRG neurons, where the mRNA expression levels were quantified at each of the three age points. Interestingly, IB4+ neurons were found to express a higher number of genes significantly affected by age than IB4− neurons. Specifically, for IB4+ neurons, RNA‐seq yielded **147** predicted differentially expressed (DE) genes from 1 to 8 months, **150** genes from 8 to 18 months, and **103** from 1 to 18 months (Figure 3a). In IB4− neurons, we found **133** predicted DE genes from 1 to 8 months, **52** genes from 8 to 18 months, and **49** from 1 to 18 months (Figure 3b). The predicted DE genes include those encoding proteins implicated in nociception and neuronal excitability, such as voltage‐gated channels permeant to K^+^ (Kv1.1, TASK1, Kv7.3), Na^+^ (Nav1.1, Nav1.7 and Nav1.9), and Ca^2+^ (Cav3.2), the ligand‐gated channels *TRPV1* and *P2RX3* and the mechanosensitive *Piezo1* and *Piezo2* channels as well as G protein‐coupled receptors *CGRP1*,* NPY1R,* and *NPY2R*. We note that a number of the DE genes related to the Gene Ontology (GO) term “aging” (GO: 0007568). Again, that number was higher within the IB4+ group (**9** in 1 to 8 months, **8** in 8 to 18 months, and **10** in 1 to 18 months), than in IB4− group (**5** in 1 to 8 months, **3** in 8 to 18 months, and **3** in 1 to 18 months; Table S1). Only four of these genes overlapped between IB4+ and IB4− neurons (*Apoe*,* Igfbp2, Phox2a, and Npy2r*). The above results again point to more pronounced age‐dependent alterations in IB4+ neurons than in IB4− ones, which is in agreement with our excitability data (Figures 1 and 2). We were therefore also interested to see whether any of these DE genes displayed similar temporal patterns of expression to the one observed in our electrophysiology experiments, where the most pronounced changes occurred around 8 months. To this end, we identified genes that were DE in periods both from 1 to 8 months and from 8 to 18 months.

**Figure 4:**
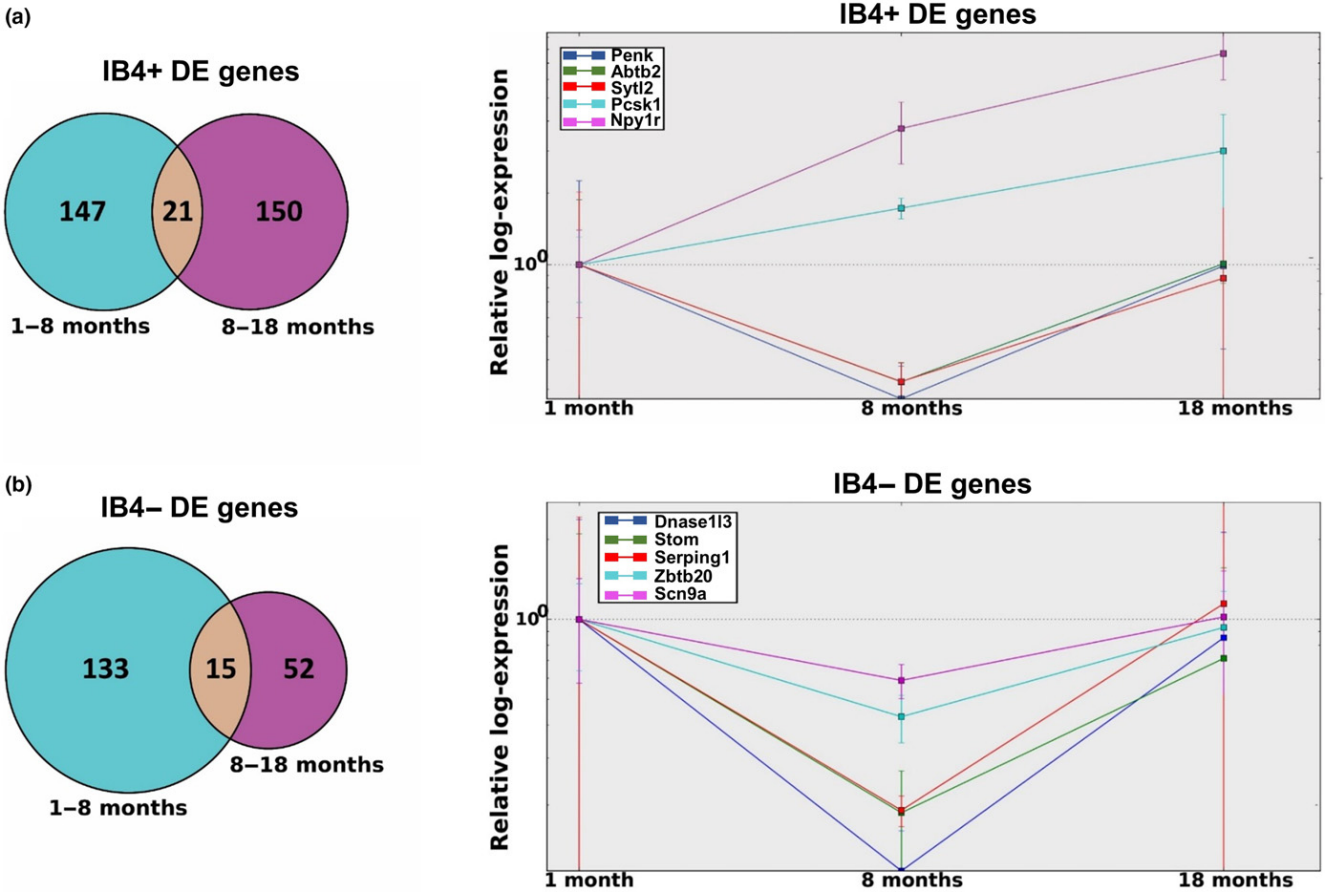
Relative log‐expression levels at 8 and 18 months reveal recovery patterns for a number of genes. (a) Left panel: 21 IB4+ genes are predicted to be differentially expressed (*p* ≤ 0.01 for all predictors) from 1 to 8 months and again from 8 to 18 months. Right panel: Five genes with the highest expression levels are presented. Npy1r and Pcsk1 are predicted to increase across all time periods, whereas PENK, Abtb2, and Sytl2 decrease from 1 to 8 months, but return close to their original expression levels by 18 months. (b) Left panel: 15 IB4− genes predicted to be differentially expressed from 1 to 8 months and again from 8 to 18 months. Right panel: Again, five genes with the highest expression levels are presented. Expression levels of all the genes decrease significantly between 1 and 8 months, but return close to their original expression levels by 18 months

This analysis yielded 21 genes in IB4+ group and 15 genes in IB4− group (Figure 4a,b, left panels). Using the 1‐month time period as a reference, we used estimated fold change values, from 1 to 8 months and from 8 to 18 months, to track each gene's predicted expression level during these periods. For clarity, and to decrease the risk of false positive events, the top five genes with highest expression counts from each group are presented in Figure 4a,b, right panels (the full set of genes is presented in Figure S1a–d, and Table S2).

For IB4+ neurons, three of these DE genes, *PENK* (precursor of enkephalin, an endogenous analgesic), *Abtb2* (protein metabolic processes, including ubiquitination), and *Sytl2* (vesicle transport), exhibit clear patterns of differential expression from 1 to 8 months and an inverse pattern from 8 to 18 months, at which point expression returns close to 1‐month levels; whereas levels of *Pcsk1* (prohormone convertase) and *Npy1r* (mediates function of neuropeptide Y) increase steadily across the three age points. For IB4− neurons, the five highest expressing DE genes, *Dnase1l3* (endonuclease with DNA hydrolytic activity), *Stom* (regulates channel activity, including ASIC channels), *Serping1* (serine protease inhibitor and kinin pathway regulator), *Zbtb20* (transcription factor, regulates certain TRP channels expression), and *Scn9a* (voltage‐gated Na^+^ channel Nav1.7, sensory neuron excitability), exhibit the same “U” pattern of expression.

### 2.3 Na^+^ and K^+^ channel mRNA expression levels are differently affected by age

Given our findings on the age‐dependent differences in excitability, particularly in IB4+ neurons (Figures 1 and 2)*,* we were particularly interested in investigating the expression pattern of K^+^ and Na^+^ channels, as these proteins are major determinants of both firing and passive membrane properties of neurons.

**Figure 5:**
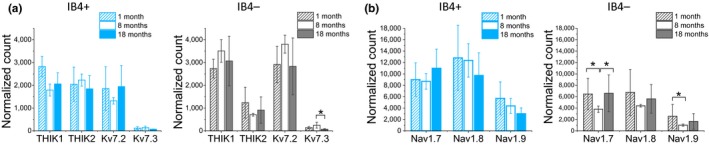
mRNA expression levels of key K^+^ and Na^+^ channel‐encoding genes in dorsal root ganglia neurons from 1‐, 8‐, and 18‐month‐old mice. (a) mRNA expression levels of THIK1, THIK2, Kv7.2, and Kv7.3 for IB4+ (left panel) and IB4− (right panel) neurons at the three age points. (b) Expression levels of Nav1.7‐, Nav1.8‐, and Nav1.9‐encoding genes for IB4+ (left panel) and IB4− (right panel) neurons. Units are corrected RPKM values

Two particular classes of K^+^ channels, encoded by the *KCNK* and *KCNQ* gene families, have previously been reported to heavily influence the RMP of rodent DRG neurons at physiological temperature (Du et al., 2014). Our RNA sequencing data revealed that the highest expressing channels from these families included *KCNK13* (THIK1), *KCNK12* (THIK2), *KCNQ2* (Kv7.2), and *KCNQ3* (Kv7.3). Interestingly, there were obvious age‐dependent differences between IB4+ and IB4− neurons with respect to the two highest expressing channels—THIK1 and Kv7.2, where the mRNA levels in neurons from 8‐month‐old mice were 53% (THIK1) and 33% (Kv7.2) lower in IB4+ than in IB4− neurons (Figure 5a). A decrease in the expression of these channels could be expected to have a depolarizing effect, which indeed was observed in our current‐clamp experiments (Figure 2a).

The expression pattern of genes encoding the three main Na^+^ channels determining excitability of DRG neurons, *Scn9a* (Nav1.7), *Scn10a* (Nav1.8), and *Scn11a* (Nav1.9) (Rush, Cummins, & Waxman, 2007), also revealed major differences between the two groups of neurons. Two noteworthy findings emerged as follows: First, that the expression levels of all three isoforms are higher in IB4+ than IB4− neurons across all age groups (Figure 5b); and second, that the expression levels of all three isoforms decrease in IB4−, but not IB4+ neurons, from 8‐month‐old mice. The observed “U” pattern of expression within the IB4− group might contribute to the observed differences in excitability between IB4+ and IB4− neurons, and particularly to the lack of an increase in repetitive firing rate in IB4− neurons from 8‐month‐old mice as observed in the IB4+ group.

### 2.4 IB4+ neurons from 8‐month‐old mice have larger TTX‐resistant window current at RMP

As repetitive firing in DRG neurons is driven primarily by the TTX‐resistant (TTX‐R) Na^+^ current (Blair & Bean, 2002; Renganathan, Cummins, & Waxman, 2001), we investigated whether there are any differences in the level of TTX‐R window current. Window currents are produced by the overlap of activation and inactivation curves and, when generated around resting membrane potentials (RMPs), indicate that persistent noninactivating current will be generated at rest, where it can modulate excitability (Crill, 1996; Vasylyev, Han, Zhao, Dib‐Hajj, & Waxman, 2014).

**Figure 6:**
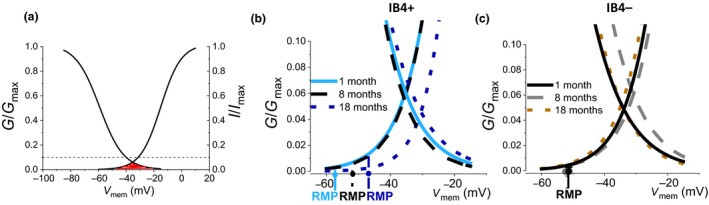
Age dependence of the TTX‐R window current in IB4+ and IB4− dorsal root ganglia neurons. (a) An example of window current (red‐colored area) produced by the overlap of activation and inactivation curves for the TTX‐R current (IB4+ neuron, 1‐month‐old mouse). The dashed line highlights the level at which the Y axes are truncated in panels b and c. (b) Window current for the TTX‐R current in IB4+ neurons from 1‐month (continuous line), 8‐month (dashed line), and 18‐month‐old (dotted line) mice. Emphasized on the *x* axis are resting membrane potentials (RMP) for the three groups of cells. (c) Window current plots for the TTX‐R current in IB4− cells from 1‐month (continuous line), 8‐month (dashed line), and 18 month‐old (dotted line) mice. RMP is the same for cells from 1‐ and 18‐month‐old mice. *N* numbers for activation curves: IB4+; 1 m = 11, 8 m = 21, 18 m = 18. IB4−; 1 m = 17, 8 m = 15, 18 m = 21. Steady‐state inactivation curves: IB4+; 1 m = 6, 8 m = 8, 18 m = 9. IB4− cells; 1 m = 7, 8 m = 7, 18 m = 10

The window current plots for the TTX‐R current in IB4+ (Figure 6b) and IB4− (Figure 6c) neurons indicate that different levels of persistent TTX‐R current will be generated around RMP at the 1‐month (solid line), 8‐month (dashed line), and 18‐month (dotted line) age points in both groups of neurons. Interestingly, at RMP, IB4+ DRG neurons from 8‐month‐old mice receive the greatest contribution from the TTX‐R window current. This is likely to lead to higher levels of persistent “background” current, contributing to membrane depolarization and higher excitability at this age point, as observed in our current‐clamp experiments (Figure 1).

## 3 DISCUSSION

In this study, we combined whole‐cell voltage‐ and current‐clamp measurements at physiological temperature and RNA‐seq techniques to examine age‐dependent alterations in neurophysiology and gene transcription of murine IB4+ and IB4− DRG neurons. We found that IB4+ neurons are subject to more pronounced age‐dependent changes than IB4− neurons. Particularly noteworthy was the gradual increase in excitability of IB4+ neurons from 1 to 8 months followed by a decrease from 8 to 18 months, as firing frequency of DRG neurons has been shown to be strongly linked to pain mechanisms (Devor, 2006; Zhang et al., 2013).

The increase in firing rate in IB4+ neurons from 8‐month‐old mice appears to be due, at least in part, to a combination of significantly more depolarized RMP and high Ri. The depolarization of the membrane potential of 8‐month‐old IB4+ neurons is important, as it nears the activation potentials of the TTX‐R Nav1.8 isoform (~−40 mV), which was shown to be the main Na^+^ channel isoform underlying the majority of the current during AP upstroke and key to enabling repetitive firing in mouse DRG neurons (Akopian et al., 1999; Renganathan et al., 2001). Indeed, we found that the IB4+ neurons from 8‐month‐old animals receive the highest contribution from the TTX‐R window current at rest (Figure 6b). This TTX‐R current is likely to be a contributor to the increased excitability in IB4+ neurons from 8‐month‐old mice at depolarized membrane potentials (Figures 1 and 2). It is feasible that this window current contributes to RMP depolarization observed in IB4+ neurons from 8‐month‐old mice, as persistent TTX‐R Na^+^ current has already been shown to cause membrane depolarization of DRG neurons (Herzog et al., 2001). On the other hand, the decrease in mRNA expression for the three main Na^+^ channel isoforms underlying DRG excitability in IB4− neurons from 8‐month‐old mice (Figure 5b) may contribute to the lack of an increase in excitability around this age point.

Interestingly, we found that at the transcriptome level, IB4+ neurons from 8‐month‐old mice also appeared to be subject to more age‐dependent alterations, with a higher number of DE genes than in IB4− neurons. It is particularly interesting that in IB4+ neurons from this age group there is a significant decrease in the mRNA expression of PENK, low levels of which have been implicated in pronociceptive states (Minett et al., 2015). Combined with the observed increase in excitability, it is likely that IB4+ neurons from middle‐aged mice are particularly vulnerable to proalgesic inputs. Conversely, the decrease in the mRNA expression of Scn9a (an established pain target), Zbtb20 (regulation of TRPV1, TRPA1, TRP8M expression), and Stom (regulation of ASIC2 and ASIC3 channel activity and mechanosensitivity), could result in a decreased nociceptive responsiveness of IB4− neurons around this age point.

We also note that, unexpectedly, our results show an apparent increase in the mRNA levels for TRPV1 in IB4+ neurons from 1 to 18 months (2.8‐fold increase). While a number of studies point to a decreased thermal sensitivity with age and to low levels of TRPV1 expression in IB4+ neurons, it has been shown that peripheral inflammation selectively increases the expression and function of TRPV1 in IB4+ mouse DRG neurons (Breese, George, Pauers, & Stucky, 2005). It is therefore feasible that the reported age‐dependent increase in inflammatory states in mice (Ray, Johnston, Verhulst, Trammell, & Toth, 2010) results in the observed upregulation in TRPV1 levels in IB4+ neurons from aged mice.

From the clinical perspective, the increase in excitability of IB4+ neurons from 8‐month‐old mice is of considerable interest. In vivo studies have shown an increase in pain sensitivity in middle‐aged rats (Gagliese & Melzack, 1999) and mice (Finkel et al., 2006). Moreover, studies on human pain prevalence also point to a spike in pain‐related complaints in middle‐aged subjects (Cutler, Fishbain, Rosomoff, & Rosomoff, 1994; Rustoen et al., 2005). Notably, painful peripheral neuropathy, even in patients who carry causative Na^+^ channel mutations throughout life, usually begins to produce symptoms in middle age (Faber et al., 2012; Huang et al., 2014). In addition, middle‐aged patients tend to report more intensive psychological impacts of their conditions and greater pain “unpleasantness” levels (Gibson & Lussier, 2012; Rustoen et al., 2005). The information carried by mouse IB4+ neurons has been shown to be conveyed to the bed nucleus of the stria terminalis, which is involved in persistent anxiety responses, and to the globus pallidus, which is involved in motor control (Braz et al., 2005). This pathway was shown to be independent of the one utilized by IB4− neurons. While live human DRG neurons (hDRGs) were not found to bind IB4, immunohistochemical studies do confirm IB4 staining both in human (Pan et al., 2012; Shi et al., 2008) and primate DRGs (Gerke & Plenderleith, 2002). Moreover, a microneurography study found that mechano‐responsive fibers, which are predominantly IB4+ in mice, constitute 59% and 40% of human C‐fibers in young and older subjects, respectively (Namer et al., 2009). It is therefore feasible to speculate that if the corresponding group of neurons in hDRGs also displays an increase in excitability in middle‐aged subjects, it may contribute to the reported increase in pain sensitivity and altered affective aspect of pain within the middle‐aged population, which warrants further investigation.

It should be noted that mouse DRGs are an extremely heterogenous group of neurons, and it is likely that there are subgroups within both IB4+ and IB4− neurons that follow different temporal patterns of aging. Additionally, a study has found more pronounced age‐dependent neuropeptide expression changes in lumbar than cervical rat DRG neurons (Bergman et al., 1996), pointing to pronounced anatomical differences. It should also be acknowledged that this study did not include mice of very old age (up to ~30 months (Jonker et al., 2013); there are difficulties associated with senescent animals, including issues with both survival rate and cost of either obtaining or aging mice to that point. Future studies will also be needed to assess the effect of aging on the proteome of IB4+ and IB4− DRG neurons. Nevertheless, this study provides valuable insight into the impact of aging on two groups of DRG neurons that were shown to underlie different types of pain, and contributes to a mechanistic understanding of age‐related alterations in the excitability and transcriptome of DRG neurons. These results also suggest that age‐related changes in analgesic efficacy may be due to not only altered metabolic processes, but also to the neurophysiological and transcriptomic changes at the nociceptor level, and highlight the potential for targeting a particular group of nociceptors in age‐adjusted pharmacological therapies for the treatment of pain.

## 4 EXPERIMENTAL PROCEDURES

### 4.1 Animals

Male C57BL/6J mice of six ages were used as follows: 1 ± 0.1 month, 3 ± 0.1 months, 5 ± 0.1 months, 8 ± 0.3 months, 12 ± 0.3 months, and 18 ± 0.3 months. These are considered to correspond to prepubescent (1 m), young adult (3 m and 5 m), middle (8 m, 12 m), and old (18 m) ages in mice of this strain (Goodrick, 1975). A minimum of four animals were used to obtain any one data set. All experiments were conducted in accordance with the UK Animals (Scientific Procedures) Act 1986.

### 4.2 Cell culture

The DRG culture method was adapted from Passmore (2005). Briefly, C57Bl/6J mice of an appropriate age were killed by cervical dislocation and decapitated, the spinal column was immediately removed, and any excess tissue trimmed off, the spinal cord was removed and the exposed DRGs were collected from all levels and immersed in Lebovitz L‐15 Glutamax media. After removing spinal and dorsal roots, DRGs were incubated for 15 min at room temperature in L‐15 media containing 1 mg/ml collagenase type 1A and 6 mg/ml bovine serum albumin (BSA) and then rinsed thoroughly in Hank's balanced salt solution (HBSS). Next, the cells were incubated for 30 min at 37°C in 1 mg/ml trypsin and 6 mg/ml BSA. The cells were then manually dissociated and plated on coverslips coated with poly‐L‐lysine. After a 2‐hr adhesion period, DRGs were flooded with 2 ml of L‐15 media supplemented with 10% FBS, 24 mM NaHCO3, 2%–3% penicillin‐streptomycin, and 38 mM glucose. Cultures were maintained for up to 48 hrs under standard cell culture conditions. All media and supplements were obtained from Sigma‐Aldrich (Dorset, UK) and Invitrogen (Paisley, UK). IB4+ DRG neurons were marked with fluorescein isothiocyante‐conjugated isolectin B4 (IB4‐FITC; Invitrogen). To achieve this, cells were incubated for 15 min in external recording solution containing 2.5 μg/ml labeled IB4. This method of identifying IB4+ neurons was shown not to affect the biophysical properties or health of DRG neurons in multiple studies (Dailey & Waite, 1999; Stucky & Lewin, 1999). Cells were rinsed three times before recordings. Fluorescently labeled cells were visualized using FITC filters incorporated into the recording microscope set‐up. IB4− cells were identified as small‐diameter (<25 μm) neurons with complete resistance to IB4‐FITC staining.

### 4.3 Electrophysiology

The cells were continuously perfused (2–3 ml/min) with external recording solution consisting of (mM): NaCl, 135; KCl, 3; HEPES‐NaOH, 10; MgCl2, 1; CaCl2, 2; D‐glucose, 30; pH 7.3, 300–310 mOsm. All components were obtained from Sigma‐Aldrich (Dorset, UK). Whole‐cell recordings in voltage‐ and current‐clamp configuration were obtained and amplified using an Axon MultiClamp 700A amplifier (Molecular Devices, USA). Data were digitized via an analogue to digital converter Digidata 1440a (Molecular Devices, USA) and stored on a personal computer using pClamp 10.4 software (Molecular Devices, USA), which was also used to define and execute protocols. The data were filtered at 10 kHz in voltage‐clamp and 5 kHz in current‐clamp and acquired at 100 and 50 kHz, respectively. Electrodes used for the recordings had resistances of 1–2 MΩ, when filled with internal solutions. Pipette offset and capacitance transients were canceled using the amplifier's built‐in circuitry. Access resistance was monitored throughout the duration of the recordings and a change of >20% resulted in the termination of an experiment. The pipette solution for current‐clamp experiments contained (mM): K‐gluconate 130; KCl, 20; HEPES, 10; EGTA, 0.2; Na‐GTP, 0.3; Mg‐ATP, 4 (liquid junction potential = 15 mV); for voltage‐clamp experiments, the following solution was used (mM): CsMeSO4, 130; NaCl, 20; HEPES, 10; EGTA, 0.2; Na‐GTP, 0.3; Mg‐ATP, 4 (liquid junction potential = 10 mV). The pH of each solution was adjusted to 7.3 and osmolarity to 290–300 mOsm. The calculated liquid junction potentials were corrected for arithmetically during offline analysis. Recordings were conducted at 35 ± 0.5°C. Temperature was controlled through the feedback control system of Visual Imaging and Patching Chamber 7800 (Campden Instruments). Recorded data were processed offline using pClamp v10.4 (Molecular Devices, USA), Origin v9.1 (OriginLab Co., US), Excel (Microsoft Co., USA), and custom‐written scripts in Matlab program (MathWorks, UK).

#### 4.3.1 Voltage‐clamp recordings

Isolated cells with minimal processes were chosen for recordings. After establishing the whole‐cell configuration, cells were allowed to equilibrate for 5 min. TTX‐resistant Na^+^ currents were isolated by the inclusion of 500 nM TTX (Alomone Labs, Jerusalem, Israel) in the external solution. Series resistance compensation was set at 80%–90%. The linear leak current was corrected online using the P/4 procedure (Bezanilla & Armstrong, 1977). Both raw and leak‐subtracted data were acquired. Cells with leak current >0.3 nA, access resistance of >4 MΩ, and/or current amplitude of <0.5 nA were discarded.

Activation curves, or conductance‐voltage (G‐V) curves, were constructed using G = I/(V_mem_–V_rev_); where G = conductance, I = current, V_mem_ = membrane potential, and V_rev_ = reversal potential calculated using the Nernst equation.

Steady‐state inactivation (SSI) data were gathered using a two‐voltage step protocol, in which 500 ms depolarizing prepulses ranging from −105 to −45 mV were followed by a 10 ms test pulse to −20 mV.

G‐V and SSI curves were fit with a single Boltzmann equation to determine voltage dependence of activation and inactivation: *y* = (A1−A2)/(1 + e(x−xo)/dx)) + A2, where A1 and A2 represent 0 and 100% current availability, x0 is the membrane potential at half maximal activation or inactivation of Na^+^ current (V½), and dx is the slope factor.

#### 4.3.2 Current‐clamp recordings

Cells with stable membrane potential were chosen for analysis in current‐clamp configuration. Resting membrane potential (RMP) was determined immediately after switching into current‐clamp configuration as the mean membrane voltage in the absence of current stimulation. Passive and firing properties were analyzed at both RMP and set prestimulus potentials (established by injection of bias currents of appropriate amplitudes). Input resistance (Ri) was derived from an extrapolation of a single exponential curve fit to the charging component of the voltage response to a single hyperpolarizing 50 pA step. Firing rate was determined by injecting 500 ms incremental depolarizing current steps and quantifying the number of generated action potentials (APs).

### 4.4 RNA‐seq experiments

Dorsal root ganglia cell cultures were individually prepared from four mice for each of the three age groups (i.e., a total of 12 cultures; four replicates (mice) per age point of 1, 8, and 18 months). Each culture was fluorescently labeled for IB4 in the same way as for electrophysiology. After placing cells into the patch‐clamp chamber on the fluorescent microscope, 300 IB4+ and 300 IB4− cells (<25 μm) were manually collected from each culture (one mouse) using patch‐clamp pipettes. Cells were then rapidly frozen on dry ice and then stored at −80°C. Negative controls (bath solution only) were collected for each group. RNA was extracted using the Qiagen RNeasy Micro Kit (Qiagen, USA) as per the manufacturer's protocol. Quality of RNA was assessed using Agilent 2100 Bioanalyser (Agilent Technologies, USA). RNA integrity numbers (RIN) ranged from 8.5 to 9.6. Samples were then converted to cDNA and amplified by 16 cycles according to instructions in SMARTer v3 kit (Clontech Laboratories, Inc., USA). Samples were prepared for sequencing using the Nextera XT DNA kit (Illumina, USA) and their quality assessed using the Qubit High Sensitivity DNA assay (Thermo Fisher Scientific, USA) and library quantification kits (KAPA Biosystems, USA) according to manufacturers’ instructions. Next, the libraries were pooled in equal amounts for paired‐end sequencing on an Illumina NextSeq 500 (Illumina, USA). Tophat2 (Trapnell, Pachter, & Salzberg, 2009) was used to align reads to the mouse genome (Ensembl version GRCm38) and HTSeq (Anders, Pyl, & Huber, 2015) to generate read counts from each BAM file. Several tools are available for predicting DE genes from RNA‐Seq data, among them Cuffdiff (Trapnell et al., 2013), DESeq (Anders & Huber, 2010), and edgeR (Robinson, McCarthy, & Smyth, 2010). Comprehensive reviews of these and other DE prediction methods show that while some methods (notably Cufflinks) perform poorly in simulations, no single method appears to perform consistently best (Rapaport et al., 2013; Soneson & Delorenzi, 2013; Zhang et al., 2014). Consequently, a common recommendation is to use at least two different methods and look for shared predictions. Here, both DESeq and edgeR are used (threshold of *p* < 0.01, Benjamini–Hochberg FDR adjusted). To mitigate unwanted variation, RUVSeq (Risso, Ngai, Speed, & Dudoit, 2014) was also used to adjust read counts and run edgeR a second time on the adjusted counts to yield a third set of DE scores. For our final choice of putative DE genes, the genes were ranked in ascending order using the maximum *p*‐value among the three scores for each gene. We investigated genes that could be differentially expressed (DE; up‐ or downregulated) under different conditions‐we assessed predicted DE genes between all three possible combinations of time periods: 1 versus 8 months, 8 versus 18 months, and 1 versus 18 months. The raw counts for all genes for IB4+ and IB4− groups, together with *p* values, are available in the supplementary data section (Dataset S1 and S2). The Gene Ontology (GO) assessment was performed using GOseq R Bioconductor package (Young, Wakefield, Smyth, & Oshlack, 2010) on all DE genes.

### 4.5 Statistics

Unless stated otherwise, data are presented as mean ± *SEM*,* n* refers to the number of cells. Appropriate statistical analyses were performed in origin v9.1 (OriginLab Co., USA) and spss Statistics 21 (SPSS Inc., Hong Kong). Statistical tests are stated for each individual data set in the Results section.

## CONFLICT OF INTEREST

None declared.

## References

[acel12795-bib-0001] Akopian, A. N. , Souslova, V. , England, S. , Okuse, K. , Ogata, N. , Ure, J. , … Wood, J. N. (1999). The tetrodotoxin‐resistant sodium channel SNS has a specialized function in pain pathways. Nature Neuroscience, 2, 541–548. 10.1038/9195 10448219

[acel12795-bib-0002] Anders, S. , & Huber, W. (2010). Differential expression analysis for sequence count data. Genome Biology, 11, R106 10.1186/gb-2010-11-10-r106 20979621 PMC3218662

[acel12795-bib-0003] Anders, S. , Pyl, P. T. , & Huber, W. (2015). HTSeq–a Python framework to work with high‐throughput sequencing data. Bioinformatics, 31, 166–169. 10.1093/bioinformatics/btu638 25260700 PMC4287950

[acel12795-bib-0004] Ayis, S. , Gooberman‐Hill, R. , & Ebrahim, S. (2003). Long‐standing and limiting long‐standing illness in older people: Associations with chronic diseases, psychosocial and environmental factors. Age and Ageing, 32, 265–272. 10.1093/ageing/32.3.265 12720611

[acel12795-bib-0005] Bergman, E. , Johnson, H. , Zhang, X. , Hokfelt, T. , & Ulfhake, B. (1996). Neuropeptides and neurotrophin receptor mRNAs in primary sensory neurons of aged rats. Journal of Comparative Neurology, 375, 303–319. 10.1002/(ISSN)1096-9861 8915832

[acel12795-bib-0006] Bergman, E. , & Ulfhake, B. (2002). Evidence for loss of myelinated input to the spinal cord in senescent rats. Neurobiology of Aging, 23, 271–286. 10.1016/S0197-4580(01)00274-3 11804713

[acel12795-bib-0007] Bezanilla, F. , & Armstrong, C. M. (1977). Inactivation of the sodium channel. I. Sodium current experiments. Journal of General Physiology, 70, 549–566. 10.1085/jgp.70.5.549 591911 PMC2228478

[acel12795-bib-0008] Bishay, P. , Häussler, A. , Lim, H.‐Y. , Oertel, B. , Galve‐Roperh, I. , Ferreirós, N. , & Tegeder, I. (2013). Anandamide deficiency and heightened neuropathic pain in aged mice. Neuropharmacology, 71, 204–215. 10.1016/j.neuropharm.2013.03.021 23597506

[acel12795-bib-0009] Blair, N. T. , & Bean, B. P. (2002). Roles of tetrodotoxin (TTX)‐sensitive Na+ current, TTX‐resistant Na+ current, and Ca2+ current in the action potentials of nociceptive sensory neurons. Journal of Neuroscience, 22, 10277–10290. 10.1523/JNEUROSCI.22-23-10277.2002 12451128 PMC6758735

[acel12795-bib-0010] Braz, J. M. , Nassar, M. A. , Wood, J. N. , & Basbaum, A. I. (2005). Parallel “pain” pathways arise from subpopulations of primary afferent nociceptor. Neuron, 47, 787–793. 10.1016/j.neuron.2005.08.015 16157274

[acel12795-bib-0011] Breese, N. M. , George, A. C. , Pauers, L. E. , & Stucky, C. L. (2005). Peripheral inflammation selectively increases TRPV1 function in IB4‐positive sensory neurons from adult mouse. Pain, 115, 37–49. 10.1016/j.pain.2005.02.010 15836968

[acel12795-bib-0012] Buskila, D. , Abramov, G. , Biton, A. , & Neumann, L. (2000). The prevalence of pain complaints in a general population in Israel and its implications for utilization of health services. Journal of Rheumatology, 27, 1521–1525. 10852282

[acel12795-bib-0013] Chiu, I. M. , Barrett, L. B. , Williams, E. K. , Strochlic, D. E. , Lee, S. , Weyer, A. D. , … Woolf, C. J. (2014). Transcriptional profiling at whole population and single cell levels reveals somatosensory neuron molecular diversity. Elife, 3 10.7554/eLife.04660.001 PMC4383053 25525749

[acel12795-bib-0014] Crill, W. E. (1996). Persistent sodium current in mammalian central neurons. Annual Review of Physiology, 58, 349–362. 10.1146/annurev.ph.58.030196.002025 8815799

[acel12795-bib-0015] Crisp, T. , Giles, J. R. , Cruce, W. L. , McBurney, D. L. , & Stuesse, S. L. (2003). The effects of aging on thermal hyperalgesia and tactile‐evoked allodynia using two models of peripheral mononeuropathy in the rat. Neuroscience Letters, 339, 103–106. 10.1016/S0304-3940(03)00009-0 12614905

[acel12795-bib-0016] Cutler, R. B. , Fishbain, D. A. , Rosomoff, R. S. , & Rosomoff, H. L. (1994). Outcomes in treatment of pain in geriatric and younger age groups. Archives of Physical Medicine and Rehabilitation, 75, 457–464. 10.1016/0003-9993(94)90172-4 8172508

[acel12795-bib-0017] Dailey, M. E. , & Waite, M. (1999). Confocal imaging of microglial cell dynamics in hippocampal slice cultures. Methods, 18, 222–230, 177. 10356354 10.1006/meth.1999.0775

[acel12795-bib-0018] Denton, F. T. , & Spencer, B. G. (2010). Chronic health conditions: Changing prevalence in an aging population and some implications for the delivery of health care services. Canadian Journal on Aging, 29, 11–21. 10.1017/S0714980809990390 20202262

[acel12795-bib-0019] Devor, M. (2006). Sodium channels and mechanisms of neuropathic pain. Journal of Pain, 7, S3–S12. 10.1016/j.jpain.2005.09.006 16426998

[acel12795-bib-0020] Dirajlal, S. , Pauers, L. E. , & Stucky, C. L. (2003). Differential response properties of IB(4)‐positive and ‐negative unmyelinated sensory neurons to protons and capsaicin. Journal of Neurophysiology, 89, 513–524. 10.1152/jn.00371.2002 12522198

[acel12795-bib-0021] Du, X. , Hao, H. , Gigout, S. , Huang, D. , Yang, Y. , Li, L. , … Gamper, N. (2014). Control of somatic membrane potential in nociceptive neurons and its implications for peripheral nociceptive transmission. Pain, 155, 2306–2322. 10.1016/j.pain.2014.08.025 25168672 PMC4247381

[acel12795-bib-0022] Faber, C. G. , Hoeijmakers, J. G. J. , Ahn, H.‐S. , Cheng, X. , Han, C. , Choi, J.‐S. , … Merkies, I. S. J. (2012). Gain of function Nanu1.7 mutations in idiopathic small fiber neuropathy. Annals of Neurology, 71, 26–39. 10.1002/ana.22485 21698661

[acel12795-bib-0023] Farrell, M. J. (2012). Age‐related changes in the structure and function of brain regions involved in pain processing. Pain Medicine, 13(Suppl 2), S37–S43. 10.1111/j.1526-4637.2011.01287.x 22497746

[acel12795-bib-0024] Finkel, J. C. , Besch, V. G. , Hergen, A. , Kakareka, J. , Pohida, T. , Melzer, J. M. , … Quezado, Z. M. N. (2006). Effects of aging on current vocalization threshold in mice measured by a novel nociception assay. Anesthesiology, 105, 360–369. 10.1097/00000542-200608000-00020 16871071 PMC4780048

[acel12795-bib-0025] Gagliese, L. , & Melzack, R. (1999). Age differences in the response to the formalin test in rats. Neurobiology of Aging, 20, 699–707. 10.1016/S0197-4580(99)00061-5 10674437

[acel12795-bib-0026] Gerke, M. B. , & Plenderleith, M. B. (2002). Analysis of the distribution of binding sites for the plant lectin Bandeiraea simplicifolia I‐isolectin B4 on primary sensory neurones in seven mammalian species. Anatomical Record, 268, 105–114. 10.1002/(ISSN)1097-0185 12221716

[acel12795-bib-0027] Gibson, S. J. , & Lussier, D. (2012). Prevalence and relevance of pain in older persons. Pain Medicine, 13(Suppl 2), S23–S26. 10.1111/j.1526-4637.2012.01349.x 22497744

[acel12795-bib-0028] Goodrick, C. L. (1975). Life‐span and the inheritance of longevity of inbred mice. Journal of Gerontology, 30, 257–263. 10.1093/geronj/30.3.257 1120887

[acel12795-bib-1000] Herzog, R. I. , Cummins, T. R. , Waxman, S. G. (2001). Persistent TTX‐resistant Na+ current affects resting potential and response to depolarization in simulated spinal sensory neurons. J Neurophysiol 86, 1351–1364. 11535682 10.1152/jn.2001.86.3.1351

[acel12795-bib-0029] Huang, J. , Han, C. , Estacion, M. , Vasylyev, D. , Hoeijmakers, J. G. J. , Gerrits, M. M. , … Waxman, S. G. (2014). Gain‐of‐function mutations in sodium channel Na(v)1.9 in painful neuropathy. Brain, 137, 1627–1642. 10.1093/brain/awu079 24776970

[acel12795-bib-0030] Jonker, M. J. , Melis, J. P. M. , Kuiper, R. V. , van der Hoeven, T. V. , Wackers, P. F. K. , Robinson, J. , … van Steeg, H. (2013). Life spanning murine gene expression profiles in relation to chronological and pathological aging in multiple organs. Aging Cell, 12, 901–909. 10.1111/acel.12118 23795901 PMC3772962

[acel12795-bib-0031] Lovell, J. A. , Novak, J. C. , Stuesse, S. L. , Cruce, W. L. , & Crisp, T. (2000). Changes in spinal serotonin turnover mediate age‐related differences in the behavioral manifestations of peripheral nerve injury. Pharmacology, Biochemistry and Behavior, 66, 873–878. 10.1016/S0091-3057(00)00285-9 10973528

[acel12795-bib-0032] Malmberg, A. B. , Chen, C. , Tonegawa, S. , & Basbaum, A. I. (1997). Preserved acute pain and reduced neuropathic pain in mice lacking PKCgamma. Science, 278, 279–283. 10.1126/science.278.5336.279 9323205

[acel12795-bib-0033] Mantyh, P. W. , Rogers, S. D. , Honore, P. , Allen, B. J. , Ghilardi, J. R. , Li, J. , … Simone, D. A. (1997). Inhibition of hyperalgesia by ablation of lamina I spinal neurons expressing the substance P receptor. Science, 278, 275–279. 10.1126/science.278.5336.275 9323204

[acel12795-bib-0034] Minett, M. S. , Pereira, V. , Sikandar, S. , Matsuyama, A. , Lolignier, S. , Kanellopoulos, A. H. , … Wood, J. N. (2015). Endogenous opioids contribute to insensitivity to pain in humans and mice lacking sodium channel Nav1.7. Nature Communications, 6, 8967 10.1038/ncomms9967 PMC4686868 26634308

[acel12795-bib-0035] Namer, B. , Barta, B. , Ørstavik, K. , Schmidt, R. , Carr, R. , Schmelz, M. , & Handwerker, H. O. (2009). Microneurographic assessment of C‐fibre function in aged healthy subjects. Journal of Physiology, 587, 419–428. 10.1113/jphysiol.2008.162941 19064617 PMC2670053

[acel12795-bib-0036] Pan, A. , Wu, H. , Li, M. , Lu, D. , He, X. , Yi, X. , … Li, Z. (2012). Prenatal expression of purinergic receptor P2X3 in human dorsal root ganglion. Purinergic Signalling, 8, 245–254. 10.1007/s11302-011-9277-0 22052556 PMC3350579

[acel12795-bib-0037] Passmore, G. M. (2005). Dorsal root ganglion neurones in culture: A model system for identifying novel analgesic targets? Journal of Pharmacological and Toxicological Methods, 51, 201–208. 10.1016/j.vascn.2004.08.007 15862465

[acel12795-bib-0038] Rapaport, F. , Khanin, R. , Liang, Y. , Pirun, M. , Krek, A. , Zumbo, P. , … Betel, D. (2013). Comprehensive evaluation of differential gene expression analysis methods for RNA‐seq data. Genome Biology, 14, R95 10.1186/gb-2013-14-9-r95 24020486 PMC4054597

[acel12795-bib-0039] Ray, M. A. , Johnston, N. A. , Verhulst, S. , Trammell, R. A. , & Toth, L. A. (2010). Identification of markers for imminent death in mice used in longevity and aging research. Journal of the American Association for Laboratory Animal Science, 49, 282–288. 20587157 PMC2877298

[acel12795-bib-0040] Renganathan, M. , Cummins, T. R. , & Waxman, S. G. (2001). Contribution of Na(v)1.8 sodium channels to action potential electrogenesis in DRG neurons. Journal of Neurophysiology, 86, 629–640. 10.1152/jn.2001.86.2.629 11495938

[acel12795-bib-0041] Risso, D. , Ngai, J. , Speed, T. P. , & Dudoit, S. (2014). Normalization of RNA‐seq data using factor analysis of control genes or samples. Nature Biotechnology, 32, 896–902. 10.1038/nbt.2931 PMC4404308 25150836

[acel12795-bib-0042] Robinson, M. D. , McCarthy, D. J. , & Smyth, G. K. (2010). edgeR: A Bioconductor package for differential expression analysis of digital gene expression data. Bioinformatics, 26, 139–140. 10.1093/bioinformatics/btp616 19910308 PMC2796818

[acel12795-bib-0043] Rush, A. M. , Cummins, T. R. , & Waxman, S. G. (2007). Multiple sodium channels and their roles in electrogenesis within dorsal root ganglion neurons. Journal of Physiology, 579, 1–14. 10.1113/jphysiol.2006.121483 17158175 PMC2075388

[acel12795-bib-0044] Rustoen, T. , Wahl, A. K. , Hanestad, B. R. , Lerdal, A. , Paul, S. , & Miaskowski, C. (2005). Age and the experience of chronic pain: Differences in health and quality of life among younger, middle‐aged, and older adults. Clinical Journal of Pain, 21, 513–523. 10.1097/01.ajp.0000146217.31780.ef 16215337

[acel12795-bib-0045] Schopflocher, D. , Taenzer, P. , & Jovey, R. (2011). The prevalence of chronic pain in Canada. Pain Research and Management, 16, 445–450. 10.1155/2011/876306 22184555 PMC3298051

[acel12795-bib-0046] Shi, T. J. , Liu, S. X. , Hammarberg, H. , Watanabe, M. , Xu, Z. Q. , & Hokfelt, T. (2008). Phospholipase C{beta}3 in mouse and human dorsal root ganglia and spinal cord is a possible target for treatment of neuropathic pain. Proceedings of the National Academy of Sciences of the United States of America, 105, 20004–20008. 19066214 10.1073/pnas.0810899105 PMC2604939

[acel12795-bib-0047] Silverman, J. D. , & Kruger, L. (1990). Selective neuronal glycoconjugate expression in sensory and autonomic ganglia: Relation of lectin reactivity to peptide and enzyme markers. Journal of Neurocytology, 19, 789–801. 10.1007/BF01188046 2077115

[acel12795-bib-0048] Soneson, C. , & Delorenzi, M. (2013). A comparison of methods for differential expression analysis of RNA‐seq data. BMC Bioinformatics, 14, 91 10.1186/1471-2105-14-91 23497356 PMC3608160

[acel12795-bib-0049] Stucky, C. L. , & Lewin, G. R. (1999). Isolectin B(4)‐positive and ‐negative nociceptors are functionally distinct. Journal of Neuroscience, 19, 6497–6505. 10.1523/JNEUROSCI.19-15-06497.1999 10414978 PMC6782829

[acel12795-bib-0050] Trapnell, C. , Hendrickson, D. G. , Sauvageau, M. , Goff, L. , Rinn, J. L. , & Pachter, L. (2013). Differential analysis of gene regulation at transcript resolution with RNA‐seq. Nature Biotechnology, 31, 46–53. 10.1038/nbt.2450 PMC3869392 23222703

[acel12795-bib-0051] Trapnell, C. , Pachter, L. , & Salzberg, S. L. (2009). TopHat: Discovering splice junctions with RNA‐Seq. Bioinformatics, 25, 1105–1111. 10.1093/bioinformatics/btp120 19289445 PMC2672628

[acel12795-bib-0052] UN (2015). World population ageing 2015. United Nations, Department of Economic and Social Affairs, Population Division (ST/ESA/SER.A/390).

[acel12795-bib-0053] Vasylyev, D. V. , Han, C. , Zhao, P. , Dib‐Hajj, S. , & Waxman, S. G. (2014). Dynamic‐clamp analysis of wild‐type human Nav1.7 and erythromelalgia mutant channel L858H. Journal of Neurophysiology, 111, 1429–1443. 10.1152/jn.00763.2013 24401712

[acel12795-bib-0054] Yeo, S. N. , & Tay, K. H. (2009). Pain prevalence in Singapore. Annals of the Academy of Medicine, Singapore, 38, 937–942. 19956814

[acel12795-bib-0055] Yezierski, R. P. (2012). The effects of age on pain sensitivity: Preclinical studies. Pain Medicine, 13(Suppl 2), S27–S36. 10.1111/j.1526-4637.2011.01311.x 22497745 PMC3565621

[acel12795-bib-0056] Young, M. D. , Wakefield, M. J. , Smyth, G. K. , & Oshlack, A. (2010). Gene ontology analysis for RNA‐seq: Accounting for selection bias. Genome Biology, 11, R14 10.1186/gb-2010-11-2-r14 20132535 PMC2872874

[acel12795-bib-0057] Zhang, Z. H. , Jhaveri, D. J. , Marshall, V. M. , Bauer, D. C. , Edson, J. , Narayanan, R. K. , … Zhao, Q. Y. (2014). A comparative study of techniques for differential expression analysis on RNA‐Seq data. PLoS ONE, 9, e103207 10.1371/journal.pone.0103207 25119138 PMC4132098

[acel12795-bib-0058] Zhang, X. Y. , Wen, J. , Yang, W. , Wang, C. , Gao, L. , Zheng, L. H. , … Liu, J. Y. (2013). Gain‐of‐function mutations in SCN11A cause familial episodic pain. American Journal of Human Genetics, 93, 957–966. 10.1016/j.ajhg.2013.09.016 24207120 PMC3824123

[acel12795-bib-0059] Zylka, M. J. , Rice, F. L. , & Anderson, D. J. (2005). Topographically distinct epidermal nociceptive circuits revealed by axonal tracers targeted to Mrgprd. Neuron, 45, 17–25. 10.1016/j.neuron.2004.12.015 15629699

