# Supplementary figures and images for: Differential aging‐related changes in neurophysiology and gene expression in IB4‐positive and IB4‐negative nociceptive neurons
Source: Aging Cell. 2018 Jun 25;17(4):e12795. doi: 10.1111/acel.12795 (PMC6052481; doi:10.1111/acel.12795)

(A)

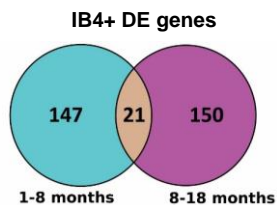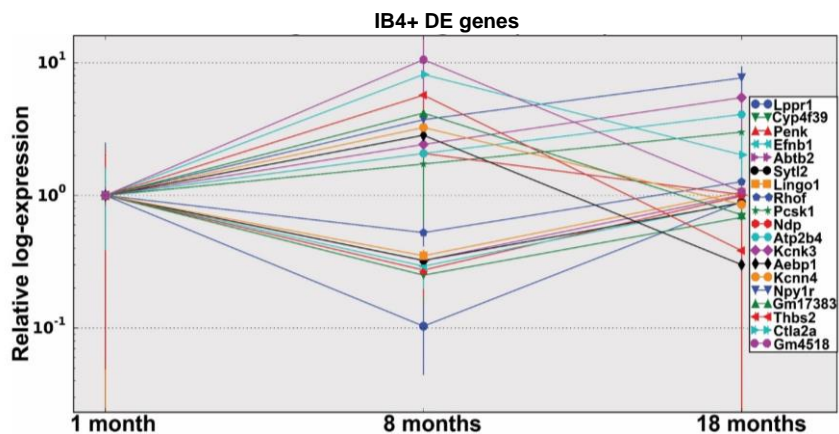

(B)

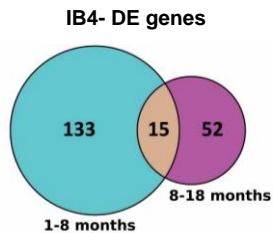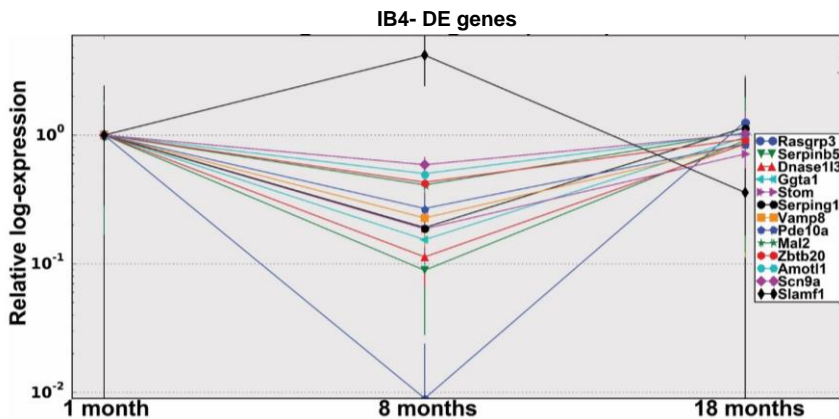

(C)

*IB4*<sup>+</sup>*1 month**8 months**18 months*

| R1 | R2 | R3 | R4 | R1 | R2 | R3 | R4 | R1 | R2 | R3 | R4 |
|----|----|----|----|----|----|----|----|----|----|----|----|
|----|----|----|----|----|----|----|----|----|----|----|----|

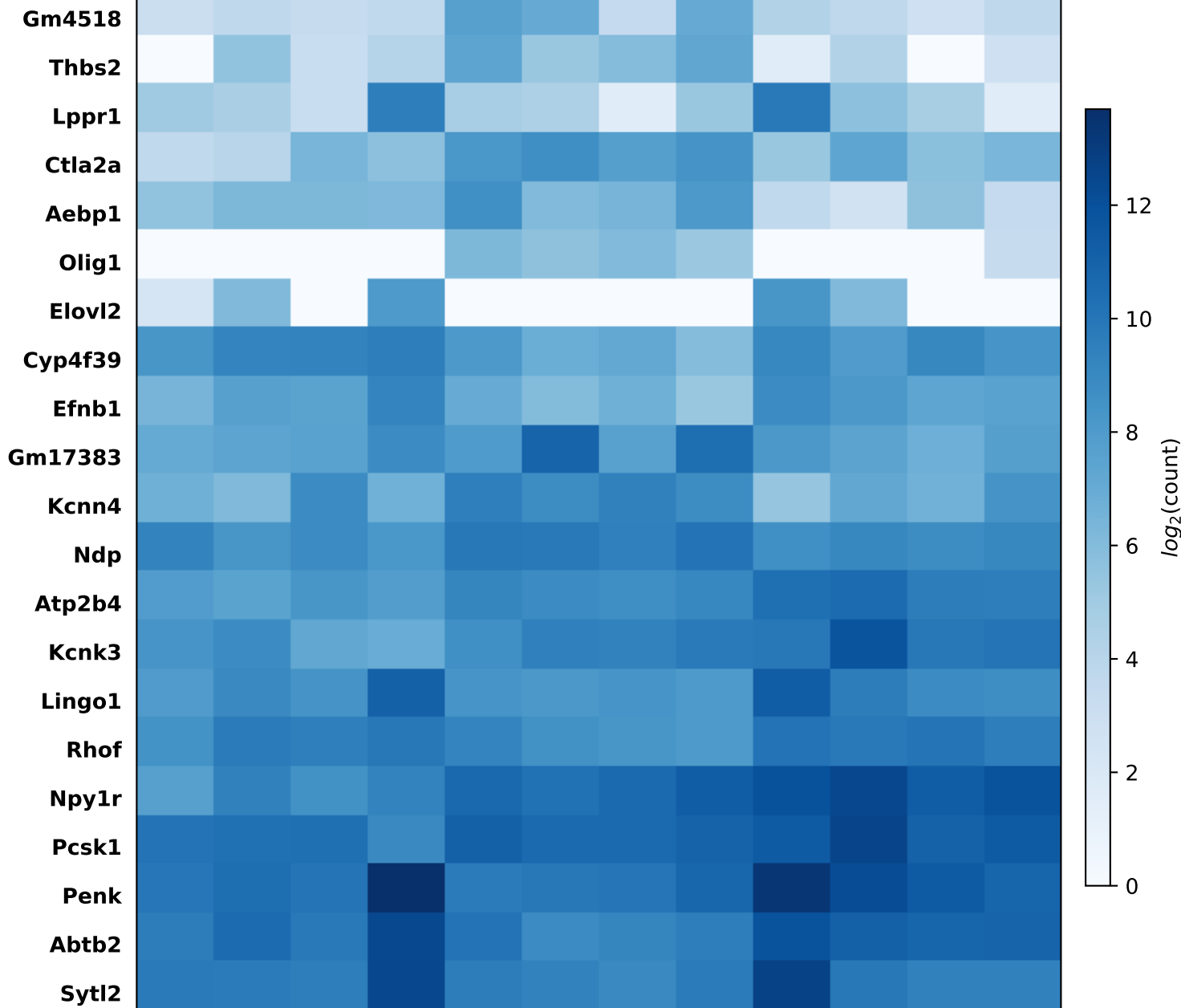

(D)

*IB4*<sup>-</sup>*1 month**8 months**18 months*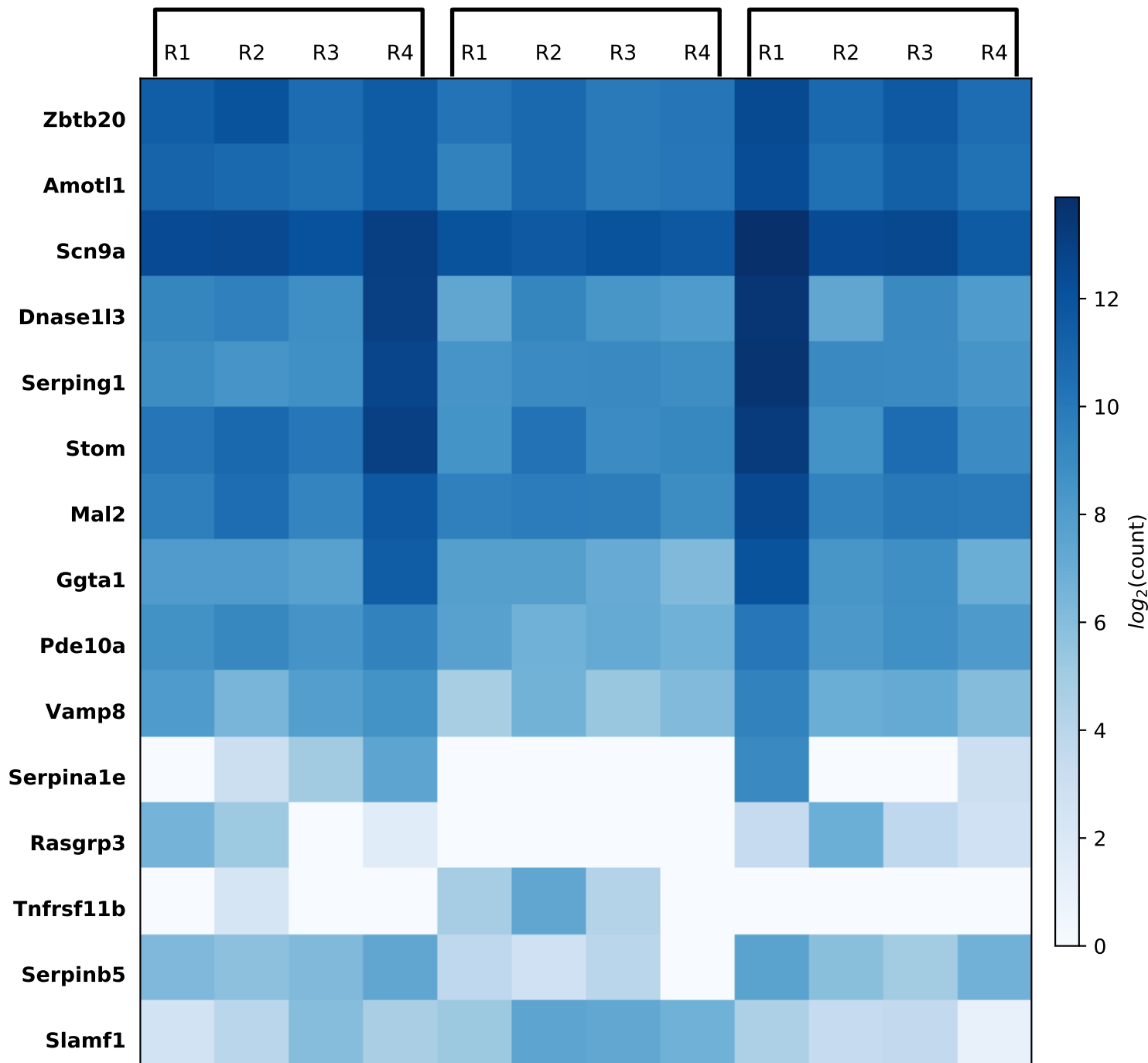

Supplement: Supplementary file 1 [file ACEL-17-na-s001.pdf]
