# Supplementary material for: Differential aging‐related changes in neurophysiology and gene expression in IB4‐positive and IB4‐negative nociceptive neurons
Source: Aging Cell. 2018 Jun 25;17(4):e12795. doi: 10.1111/acel.12795 (PMC6052481; doi:10.1111/acel.12795)
Supplement: Supplementary file 2 [file ACEL-17-na-s002.pdf]

IB4+ 1 vs. 8			IB4+ 8 vs. 18			IB4+ 1 vs. 18		
**Gene**	**Fold change**	**p value**	**Gene**	**Fold change**	**p value**	**Gene**	**Fold change**	**p value**
**Phox2a**	6.7	0.0002	Ptgs2	42	0.007	Cldn1	81.5	0.001
Npy	4	0.006	Cldn1	134	0.0003	Igfbp3	0.5	0.0001
Pth1r	3.2	0.007	Cryab	0.2	0.00002	Calca	2	0.0001
Npy1r	3.7	0.0000001	**Igfbp2**	0.4	0.001	**Apoe**	0.3	0.00001
Snca	0.2	0.00005	**Npy2r**	0.3	0.0004	Ednrb	0.3	0.0001
Mbp	0.2	0.0007	Ednrb	0.4	0.009	Npy1r	7.7	1.1E-15
Penk	0.3	0.005	Npy1r	2	0.00004	Nos1	3.1	0.007
Igfbp3	0.6	0.002	Penk	3.7	0.000003	Pth1r	3.2	0.00003
Calca	1.8	0.00004				Flt1	4.4	0.0003
						Ppp1r9a	0.2	0.0005

IB4- 1 vs. 8			IB4- 8 vs. 18			IB4- 1 vs. 18		
**Gene**	**Fold change**	**p value**	**Gene**	**Fold change**	**p value**	**Gene**	**Fold change**	**p value**
**Phox2a**	5.9	0.005	Serping1	5.9	0.001	**Npy2r**	0.2	0.003
Kdr	0.04	0.0005	Snca	0.4	0.0005	Gfra1	0.4	0.003
**Igfbp2**	0.3	0.008	**Apoe**	0.4	4.5E-09	**Apoe**	0.2	5.1E-09
Prkca	0.3	0.0005						
Serping1	0.2	0.003						
