# Supplementary material for: Differential aging‐related changes in neurophysiology and gene expression in IB4‐positive and IB4‐negative nociceptive neurons
Source: Aging Cell. 2018 Jun 25;17(4):e12795. doi: 10.1111/acel.12795 (PMC6052481; doi:10.1111/acel.12795)
Supplement: Supplementary file 3 [file ACEL-17-na-s003.pdf]

IB4+		IB4-	
**gene**	**Function**	**gene**	**Function**
**AEBP1**	Adipocyte enhancer-binding protein 1; wound healing, abdominal wall development, pro-inflammatory mediator	**Slamf1**	Signalling Lymphocytic Activation Molecule Family Member 1; signalling between immune cells, regulation of immune response
**Thbs2**	Thrombospondin 2; modulation of cell adhesion and motility	**Rasgrp3**	RAS Guanyl Releasing Protein 3; activates small GTPases including Ras and Rap1, dysregulation linked to rheumatoid arthritis
**Kcnn4**	Potassium Calcium-Activated Channel Subfamily N Member 4; Ca^{2+} transport, cell volume control, macrophage multinucleation, linked to sickle-cell and inflammatory disease	**Serpinb5**	Serpin Family B Member 5; tumour suppressor
**Ndp**	Norrin cystine knot growth factor; activates Wnt signalling pathway	**Dnase1l3**	Deoxyribonuclease I Like 3; hydrolyses DNA and mediates the breakdown of DNA during apoptosis
**Ctla2a**	Protein CTLA-2-alpha; expressed in cytotoxic T lymphocytes, immunological response	**Ggta1**	α1,3-galactosyltransferase; involved in protein glycosylation at cell surface
**Gm17383**	tubulin, alpha 1B pseudogene	**Stom**	Stomatin; regulates ion channels and transporters, including ASIC2 and ASIC3 channel activity
**Gm4518**	glyceraldehyde-3-phosphate dehydrogenase pseudogene	**Serping1**	Serpin Family G Member 1; inhibitor of the complement system; role in blood coagulation & generation of kinins
**Lppr1**	Lipid phosphate phosphatase-related protein type 1; neuronal plasticity, phospholipid metabolic processes	**Vamp8**	Vesicle-associated membrane protein 8; fusion of cellular membranes, autophagy, regulation of endo- and exocytosis
**PENK**	Preproenkephalin; precursor of Met- and Leu-enkephalin involved in modulating pain perception, suggested anti-inflammatory role	**Pde10a**	Phosphodiesterase 10A; regulates the intracellular concentration of cyclic nucleotides
**Cyp4f39**	Cytochrome P450, family 4, subfamily f, polypeptide 39; monooxygenase activity, iron ion binding, metal ion binding, oxidoreductase activity	**Mal2**	MAL Protein 2; function in transcytosis
**Efnb1**	Ephrin-B; cell-to-cell signalling, axon guidance, cell adhesion, upregulation in rheumatoid arthritis	**Zbtb20**	Zinc finger and BTB domain-containing protein 20; transcription factor, regulates TRPV1, TRPA1, TRP8M expression
**Abtb2**	Ankyrin repeat and BTB domain containing 2; regulation of protein proteolysis and ubiqutination,	**Amotl1**	Angiomotin-like protein 1; component of tight junctions, involved in maintaining cell polarity
**Sytl2**	Synaptotagmin like 2; RAB27A-dependent vesicle trafficking and secretion, required for mitochondrial fusion following cellular stress	**Scn9a**	Sodium Voltage-Gated Channel Alpha Subunit 9; crucial modulator of nociceptor excitability, mutations linked to congenital insensitivity to pain and primary inherited erythromelalgia
**Lingo1**	Leucine Rich Repeat And Ig Domain Containing 1; negative regulation of myelination by oligodendrocytes		
**Rhof**	Ras Homolog Family Member F (In Filopodia); actin filament organisation, formation of filopodia		
**Pcsk1**	Proprotein Convertase Subtilisin/Kexin Type 1; proteolytic activation of polypeptide hormones and neuropeptides precursors, including enkephalin and somatostatin; deficiency linked to obesity		
**Atp2b4**	ATPase Plasma Membrane Ca^{2+} Transporting 4; Ca^{2+} ATPase, intracellular Ca^{2+} homeostasis		
**Kcnk3**	Potassium Two Pore Domain Channel Subfamily K Member 3; acid-sensitive background K^+ channel, activated by isoflurane and halothane		
**Npy1r**	Neuropeptide Y receptor type 1; mediates function of neuropeptide Y and peptide YY; regulation of inflammation and pain behaviours		
